# Predictors of appropriate breastfeeding knowledge among pregnant women in Moshi Urban, Tanzania: a cross-sectional study

**DOI:** 10.1186/s13006-017-0102-4

**Published:** 2017-02-14

**Authors:** Tamara H. Hashim, Melina Mgongo, Johnson Katanga, Jacqueline G. Uriyo, Damian J. Damian, Babill Stray-Pedersen, Margareta Wandel, Sia E. Msuya

**Affiliations:** 1 Better Health for African Mother and Child, Box 8418, Moshi, Tanzania; 2 Institute of Basic Medical Sciences, Faculty of Medicine, University of Oslo, Oslo, Norway; 3 Institute of Clinical Medicine, Faculty of Medicine, University of Oslo, Oslo, Norway; 4 Ocean Road Cancer Institute, Directorate of Cancer Prevention Services, PO Box 3592, Dar es Salaam, Tanzania; 5 Kilimanjaro Christian Medical University College (KCMUCo), PO Box 2240, Moshi, Tanzania; 6 Division of Gynaecology & Obstetrics, Oslo University Hospital, University of Oslo, Oslo, Norway; 7 Community Health Department, KCMC Hospital, Po Box 3010, Moshi, Tanzania; 8 Department of Epidemiology and Biostatistics, Institute of Public Health, Kilimanjaro Christian Medical University College (KCMU Co), Box 2240, Moshi, Tanzania

**Keywords:** Breastfeeding knowledge, Infant feeding, Exclusive breastfeeding, Optimal breastfeeding practices, Predictors, Tanzania

## Abstract

**Background:**

Knowledge on infant feeding among pregnant women is essential when promoting optimal breastfeeding practices. This study aimed to assess the knowledge of women on optimal breastfeeding during pregnancy and associated factors as well as performance of the health system in reaching women with information on breastfeeding and infant feeding issues.

**Methods:**

A cross-sectional study was conducted from October 2013 to April 2014 among pregnant women, in their third trimester, attending for routine care at two primary health care facilities in Moshi urban, northern Tanzania.

**Results:**

A total of 536 women were enrolled, with mean age of 25.9 (SD 5.7) years. Only 51% (*n* = 274) reported to have received counselling on breastfeeding from their healthcare providers during the current pregnancy.

More than seven out of ten pregnant women were knowledgeable about key issues regarding appropriate breastfeeding practices: importance of colostrum (95%), time of breastfeeding initiation (71%), exclusive breastfeeding (EBF) (81%), and time of introducing complementary feeding (83%).

Receiving counselling on breastfeeding during the current pregnancy (Adjusted Odds Ratio [AOR] 3.7; 95% Confidence Interval [CI]: 2.4, 5.7), having two children (AOR 2.6; 95% CI: 1.5, 4.4), having three or more children (AOR 3.5; 95% CI: 1.8, 6.9) and intention to breastfeed the child exclusively (AOR 3.6; 95% CI: 2.0, 6.5) were significantly associated with appropriate breastfeeding knowledge.

**Conclusions:**

The health system failed to reach the 49% of women who did not receive counselling on infant feeding. Pregnant women who had received counselling on optimal breastfeeding and women with more than one child were more likely to have knowledge of optimal breastfeeding practices.

## Background

Despite strong evidence on immediate and long term health benefits of optimal breastfeeding in children, as shown by different studies [1–11], its practice remains very low. The initiation of breastfeeding, that is breastfeeding within an hour after delivery, is not highly practiced in many countries. In Tanzania for example, breastfeeding initiation within 1 h after birth was reported to be 59% in the 2004–2005 Tanzania Demographic and Health Survey (TDHS) and 49% in the 2010 TDHS [12, 13]. In South Asia, initiation of breastfeeding within 1 h after birth is low. Studies in India, Bangladesh and Pakistan have reported early initiation of breastfeeding prevalence of 36.4, 24 and 8.5% respectively [14].

It was estimated that in 2010 only 39% of infants aged less than six months were exclusively breastfed (EBF) in developing countries, a slight increase from 33% in 1995 [15]. In Sub Saharan Africa (SSA) countries where breastfeeding is widely practiced, only 35% of infants’ are exclusively breastfed with wide variability between countries. Data from the Demographic and Health Surveys (DHS) show that the coverage of EBF is higher in Eastern Africa (range 32–63%) compared to Southern (32–36%) or West Africa (13–25%) [16]. In order to achieve the benefits of optimal breastfeeding, more emphasis is needed on transmitting knowledge to women on the practice of optimal breastfeeding.

Several studies have evaluated prevalence and the factors influencing EBF in various SSA settings among women with infants aged six months to 12 months [17–20]. Results showed knowledge of EBF and counselling on breastfeeding during pregnancy or after delivery to be important factors influencing EBF [21, 22]. A study in Central Nepal showed that with support from health care workers and family members mothers were enabled to practice successful breastfeeding [23]. However, there is limited literature on the level of knowledge on optimal breastfeeding practices among women during pregnancy and how this may influence future optimal breastfeeding practices.

This study aims to describe the level of knowledge on optimal breastfeeding practices and associated predictors among pregnant women in Moshi municipality, northern Tanzania. This area has been chosen because studies have shown that EBF is not commonly practiced, hence to find out what is really the problem. It also assessed the source of information on breastfeeding among pregnant women attending routine antenatal care at primary health centres.

## Methods

### Study design and site

This is a cross-sectional study that aimed to describe infant feeding knowledge among pregnant women and factors influencing exclusive breastfeeding and complementary feeding among women enrolled in their third trimester of pregnancy. This paper will report on factors affecting optimal breastfeeding knowledge and its source among women during pregnancy in Moshi Municipality, northern Tanzania.

Enrolment of the pregnant women was conducted between October 2013 to April 2014 and the follow up was done up to June 2015 in the two largest government primary health care clinics (PHC), Majengo and Pasua located in Moshi municipal, in Kilimanjaro region.

### Study sample

The study population included pregnant women, who were in their third trimester and attending routine care at the two clinics. Purposive sampling was used to recruit participants for the study. All pregnant women who were attending antenatal clinic from the two clinics were considered eligible for the study. Pregnant women were first informed about the study, its aims and follow up requirement. They were informed that the study will assess their knowledge of breastfeeding, and that after enrolment they will be followed up during delivery, at 7 days and then monthly up to 6 months and finally every third month up to 1 year. Those who met the inclusion criteria and who gave informed consent were invited to participate. A total of 536 pregnant women participated in the study [24].

### Data collection procedure

Face to face interviews were conducted using questionnaires to collect information on: socio-economic and demographic factors, frequency of antenatal clinic (ANC) use, infant feeding and breastfeeding counselling offered, infant feeding method preferred, knowledge about exclusive breastfeeding, knowledge of optimal breastfeeding practices, and on their perception and intention to exclusively breastfed their infants. They were also asked about the optimal time for the initiation of breastfeeding, giving of colostrum, introduction of complementary feeding and the duration of breastfeeding as recommended in Tanzania.

The local language Swahili, was used in all interviews.

### Data processing and analysis

The data were entered, cleaned and analyzed using SPSS versions 22 (SSPS, Chicago, IL, USA) [25]. Continuous data were summarized by using means and median with respective measures of dispersion, while proportions were used to summarize categorical variables. The odds ratio (OR) with its associated 95% confidence interval was used to assess the strength of association between knowledge on optimal breastfeeding practices and predictor variables. Logistic regression analysis was used to control for confounding factors.

### Categorization of knowledge of optimal breastfeeding practices

The assessment of correct knowledge on EBF was based on two variables: a) understanding that the child is to be given breast milk only (except medications), and b) this should be practised from birth up to 6 months of the infant’s life. Adequate knowledge of optimal breastfeeding practices was considered to be correct information for the following three variables: a) that breastfeeding should be initiated within 1^st^ hour after birth, b) the duration and definition of exclusive breastfeeding, and c) the appropriate age to introduce complementary feeding. The categorization was based on WHO recommendations and the definition of optimal breastfeeding practices.

### Ethical considerations

Ethical clearance was obtained from Ethics Committee of Kilimanjaro Christian Medical University College (Ethical Clearance certificate number 899) and the Regional Committees for Medical and Health Research Ethics (REK) of Norway. Permission to conduct the study at Moshi Municipal facilities was sought from the District Medical Officer of Moshi Municipal and the Heads of Majengo and Pasua Health Committees.

Written informed consent was sought from each participant before enrolment and for those who could not write, a right thumb print was used. Research subjects were identified by a code number in all of the questionnaires.

## Results

Table 1 shows the socio-demographic and reproductive health characteristics of the 536 pregnant women who met the inclusion criteria and were included in the study. The age of the participating mothers ranged from 14 to 45 years with a mean of 25.9 years (standard deviation [SD] of 5.7 years). The majority of women were married or cohabiting 89.4%, 60% had completed primary school education, 68.1% had been employed in the past 12 months and 90.5% could afford three meals a day. Most of these women were in their first or second pregnancy 35 and 32% respectively. Most women (64%) had visited the antenatal clinic 2–3 times.

**Table 1:** Socio demographic and reproductive health characteristics of the participants (*n* = 536)

Variables	Number	Percent
Age		
15–24	260	48.5
25–34	230	42.9
35–45	46	8.6
Marital status		
Married/cohabiting	479	89.4
Single/separated/divorced	57	10.6
Education		
None	13	2.4
Primary	325	60.6
Secondary and above	198	36.9
Employment		
Yes	365	68.1
No	171	31.9
Water source		
Within the compound	391	72.9
Around the village	145	27.1
Meal frequency		
One	9	1.7
Two	23	4.3
Three	485	90.5
Four and above	19	3.5
Alcohol		
No	461	86
Occasionally	70	13.1
Daily	5	0.9
No. Living children^a^		
1	173	34.5
2	92	18.4
3 and above	48	9.6
Age of last born^a^		
< 2years	32	6.4
2–4years	112	22.4
> 4years	164	32.7
Place of last delivery (*n* = 333)^b^		
Hospital	316	95
Home	17	5
ANC visits (*n* = 525)^c^		
Once	17	3.2
2–3 times	344	65.5
4 or more times	164	31.2
Gestational age (*n* = 531)		
< 28weeks	72	13.6
> 28 weeks	459	86.4

^a^The percentages here does not add to 100 because some women had miscarriages, some had still births and some lost their children and also there are those who this is their first pregnancy

^b^The percentages does not add to 100 because only women with 2 or more pregnancies had information on where did they deliver their previous child

^c^ANC visits for this pregnancy do not tally to 100 because 11 women did not have information on their ANC visits

Fifty one per cent (51%) of the 536 pregnant women reported that they had received counselling on breastfeeding or infant feeding during this current pregnancy (see Table 2). Most of the pregnant women had knowledge of giving colostrum (75%), knowledge of the initiation of breastfeeding within 1 h after delivery (70.7%), knowledge of EBF (81%) and 83% had knowledge on appropriate time to introduce complementary foods. Only 61.2% of the women were able to answer all of the three components correctly (i.e. initiation of breastfeeding within 1 h after birth, exclusive breastfeeding and knowledge on time to introduce complementary feeding) and were thus considered to be knowledgeable on optimal breastfeeding practice.

**Table 2:** Counselling during pregnancy and Knowledge of the women on different breastfeeding subjects (*n* = 536)

Variables	Number	Percent
Counselled on BF/Infant feeding		
Yes	274	51.1
No	262	48.9
Type of advice given (multiple answers were recorded)		
Positioning	151	55.1
Attachment	122	44.5
Mastitis	52	19.0
Colostrum	111	40.5
Exclusive breastfeeding	227	83.0
Knowledge on Colostrum giving		
Yes	507	94.6
No	14	2.6
Don’t know	15	2.8
Knowledge on EBF definition		
Correct definition	436	81.3
Not Correct	100	18.7
Knowledge on time to Introduce complementary feeds		
Knowledgeable	446	83.2
Not Knowledgeable	90	16.8
Knowledge on time of Breastfeeding initiation		
Within 1 h after delivery	379	70.7
After 1 h but within 24 h	32	6.0
After 24 h	3	0.6
Don’t know	122	22.8
Intention to practice Exclusive breastfeeding		
Exclusive breastfeeding	447	83.4
Other means of feeding	89	16.6
^a^Knowledge on appropriate breastfeeding practices		
Knowledgeable	328	61.2
Not knowledgeable	208	38.8

^a^Knowledge on optimal breastfeeding practice: Breastfeeding within 1 h after birth, exclusive breastfeeding for 6 months, introduction of complementary feeding at 6 months and continue breastfeeding for 2 years

**Fig. 1:**
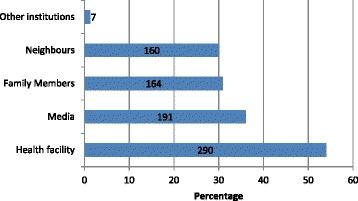
Sources of breastfeeding knowledge

The women were also asked about their sources of information on optimal breastfeeding practices. Many 54% (290) reported to have received the information from health facilities, followed by media 36.0% (191), see Fig. 1.

### Predictors of knowledge on optimal breastfeeding practices

In a bivariate logistic regression, single mothers had decreased odds of having knowledge on optimal breastfeeding practice compared to married/cohabiting mothers, Table 3. Women whose last born was older than 4 years of age also had decreased odds on knowledge about optimal breastfeeding practices (OR 0.3; 95% CI: 0.1, 0.8) compared to others. The odds of having knowledge on optimal breastfeeding practices was 2 times higher among women aged 25–49 years and 3 times higher among women with 2 or more pregnancies. The odds of having knowledge on optimal breastfeeding practices was four times higher among women who received counselling on breastfeeding or infant feeding during antenatal care attendance compared to others. Other factors like education and partner’s education were assessed but not associated with knowledge on infant feeding (see Table 3).

**Table 3:** Factors affecting appropriate knowledge on breastfeeding practices among pregnant women in Moshi urban

Variables	Number	Appropriate breastfeeding knowledge *n* (%)	OR 95% CI	AOR 95% CI
Age				
14–24	260	132 (50.8)	1	-
25–34	230	145 (63.0)	1.6 (1.2,2.4)	0.9 (0.5, 1.6)
35–49	46	31 (67.4)	2.0 (1.0, 3.9)	0.9 (0.3, 2.6)
Education				
None	13	5 (38.5)	1	-
Primary Education	325	193 (59.4)	2.3 (0.1, 7.3)	-
Secondary and higher education	198	110 (55.6)	2.0 (0.6, 6.3)	-
Marital status				
Married/cohabiting	479	285 (59.5)	1	-
Single/separated/divorced	57	23 (40.4)	0.4 (0.3, 0.8)	0.5 (0.3, 1.1)
Number of pregnancies				
1	188	71 (37.8)	1	-
2	171	113 (66.1)	3.2 (2.1, 5.0)	2.6 (1.5, 4.4)
3 and Above	177	124 (70.1)	3.9 (2.5, 6.0)	3.5 (1.8, 6.9)
Antenatal visits				
Once	17	14 (82.4)	1	-
2–3 times	344	204 (59.3)	0.3 (0.1, 1.1)	0.3 (0.1, 1.5)
4 and above	164	83 (50.6)	0.3 (0.1, 0.8)	0.2 (0.0, 1.0)
Counselling on appropriate breastfeeding practices				
Yes	274	203 (74.1)	4.3 (2.9, 6.2)	3.7 (2.4, 5.7)
No	262	105 (40.1)	1	-
Age of last born				
< 2 years	32	28 (87.5)	1	-
2–4 years	112	79 (70.5)	0.3 (0.1, 1.1)	-
> 4 years	163	108 (66.3)	0.3 (0.1, 0.8)	-
Partner’s age				
14–24	80	33 (41.3)	1	-
25–34	282	177 (62.8)	2.4 (1.4, 3.9)	1.7 (0.9, 3.2)
35–49	139	84 (60.4)	2.2 (1.2, 3.8)	0.9 (0.4, 2.2)

In multivariate logistic regression, counselling on infant feeding options (AOR 3.7; 95% CI: 2.4, 5.7), intention to practice EBF (AOR 3.6; 95% CI: 2.0, 6.5) and gravida 2 (AOR 2.6; 95% CI: 1.5, 4.4) and gravid 3 or more (AOR 3.5; 95% CI: 1.8, 6.9) remained associated with knowledge on optimal breastfeeding practices.

## Discussion

In this study it was found that at least half of the women who attended antenatal care had received counselling on optimal breastfeeding practices (51%). The results also showed that women who received counselling on infant feeding options during pregnancy and have been pregnant more than once had higher knowledge on optimal breastfeeding practices than others.

The results showed that almost half of the women who attended antenatal care had not received counselling on breastfeeding and on infant feeding (49%). This is notable, given that 96.7% of the pregnant women in this study had attended for antenatal care two or more times. The results are similar to those from Tanga in Tanzania and Northwest Ethiopia where most women attended antenatal care but few received counselling on infant feeding. In the study from Muheza district in Tanga region, 80% of the women attended for antenatal care at least once but only 39% had received counselling on infant feeding practices [22]. The study in Ethiopia showed that 78% of the women attended antenatal care at least once but only 48% had received counselling on infant feeding practices [26]. Even among those who receive counselling, the information given is suboptimal. For example only 42% of those counselled were informed about exclusive breastfeeding. Few women were counselled about attachment (23%), positioning (28%) or about breast problems like mastitis (10%) which are key when initiating and maintaining breastfeeding. The number of pregnant women that did not receive counselling on breastfeeding implies that the health system is missing the opportunity to improve women’s knowledge on appropriate infant feeding practices hence making it harder to improve the uptake and adherence to the recommended optimal breastfeeding practices. The Tanzania Focused Antenatal Care Guideline recommends that all women should receive counselling on breastfeeding as early as the first trimester [27].

In this study, counselling on breastfeeding and infant feeding during pregnancy was associated with acceptable levels of knowledge on optimal breastfeeding practices. While having knowledge on its own does not imply change in practice, several studies have shown that counselling during pregnancy not only improves knowledge, it also influences EBF practices. Studies in Kigoma and Kilimanjaro Tanzania, Zambia and Malawi have shown that women who received counselling from health providers during the antenatal or immediate postnatal periods were more likely to practice exclusive breastfeeding [20, 28, 29]. Given that 96% of pregnant women in Tanzania attended antenatal care at least once, and the finding that counselling on breastfeeding improves both knowledge and practice, health care workers should take the opportunity to educate women who attend during pregnancy and for postnatal care on breastfeeding and other key interventions to improve the health of the newborn and children. More emphasis should be put on improving counselling skills and knowledge to pregnant women as soon as they attend for antenatal care.

While only 51% of these pregnant women reported receiving counselling on breastfeeding from providers, more than 70% had knowledge of individual components of optimal breastfeeding practices. Other sources of information may have influenced their knowledge. In this study 36% (191) of the women received information on breastfeeding from the media (TV, newspapers, mobile, brochures), 30.9% (164) from close family members (mothers, sisters, mother in-law) and 30.1% (160) from neighbours. This was also found in a study by Asfaw et al. [30] in Central Ethiopia where 30.4% of women had also received information from the media and 18.4% from their friends. This shows that other sources of communication can be a good source of knowledge on appropriate feeding practices. The Ministry of Health and Social Welfare (MOHSW) should therefore develop media strategy on optimal breastfeeding practices using radio, television drama and mobile technology, with short messages as another way of imparting knowledge to the community in general and women of reproductive age on appropriate breastfeeding and infant feeding practices.

In this study, 70.7% of the women knew that breastfeeding should be initiated within 1 h. In a study done in Kilimanjaro among women with children less than 36 months, the initiation of breastfeeding within 1 h after birth was 80% [21]. Knowledge on the optimal initiation of breastfeeding probably has an influence on practice. In the TDHS 2010 report it was reported that initiation of breastfeeding within 1 h after birth in Tanzania is only 49% which has dropped from 59% in the 2004 report. This is very low and can affect the other components of optimal breastfeeding practises. Delayed initiation of breastfeeding has been found to have implications for the health of infants. Early initiation of breastfeeding within 1 h after birth has been found to reduce neonatal mortality by 22.3 and 16.3% if breastfeeding was initiated within 1 day [22]. Hence women should be taught the importance of initiating breastfeeding within 1 h as early as when they are still pregnant and soon after birth to be encouraged to start breastfeeding.

Knowledge on appropriate/optimal breastfeeding practices in this study was 61.2%, this is low given most pregnant women attended antenatal clinic more than once (96.7%). While more than 95% of Tanzanian children are breastfed for an average of 20.9 months, the other components of optimal breastfeeding practices are not performed adequately, like initiation of breastfeeding within 1 h after birth (49%), EBF (50%) or introduction of other foods and prelacteal feeding (37%) [12, 13]. It may be that the health providers are satisfied as long as the women breastfeed the children, and they forget to stress the importance of other components and their advantages in optimal growth and prevention of morbidity and mortality [2, 3, 5, 11]. There is a need to improve the training of providers on breastfeeding and child feeding issues in general. The country needs to come up with a communication strategy that will be simple and user friendly, for the women and community on breastfeeding and infant feeding practices in Tanzania. This can then be used both at facilities and in communities to contribute in improving poor breastfeeding and nutrition indicators among infants and children as a whole.

Women with three or more children were three times more likely to have knowledge on optimal breastfeeding practices than women with fewer children. This might be because the more children the woman has, the more likely she have visited the ANC more times and have received counselling every time they visit. They have also received a lot of information during postnatal visits.

### Limitations and strengths of the study

The study included pregnant women in urban Kilimanjaro hence the findings cannot be generalised for rural women in Kilimanjaro or Tanzania in general. Further, the study evaluated knowledge on optimal breastfeeding practices during pregnancy hence it is difficult to assess whether knowledge would lead to practice after delivery. Despite the weaknesses, this study is among the few studies that have addressed the knowledge on all optimal breastfeeding practices and not only exclusive breastfeeding.

## Conclusion

Based on the findings of this study we can conclude that women’s knowledge on optimal breastfeeding practice is still a low 61.5%. Ninety nine per cent of the women attend for antenatal care and 49% did not receive counselling on breastfeeding and on infant feeding practices. The study also shows that many women intended to practice EBF after delivery (83%) but few received counselling on attachment, positioning and how to deal with breast problems, an important issue to support women with their intention. Gravida and counselling were shown to have a positive influence on knowledge about optimal breastfeeding practices. Therefore emphasis should be put on making sure that every pregnant woman attending antenatal care receives counselling on appropriate infant feeding practices. Other channels of disseminating information on optimal breastfeeding practices for child growth and survival, like the media should also be used. Combining facility based and community methods of counselling might help in increasing the number of women who are aware of the importance and improve the adherence to optimal breastfeeding practices.

## Abbreviations

ANC: Antenatal care
AOR: Adjusted odds ratio
DHS: Demographic health survey
EBF: Exclusive breast feeding
PHC: Primary health care
SSA: Sub Saharan Africa
TDHS: Tanzania demographic health survey
WHO: World Health Organisation

## References

[1] Edmond KM, Zandoh C, Quigley MA, Amenga-Etego S, Owusu-Agyei S, Kirkwood BR (2006). Delayed breastfeeding initiation increases risk of neonatal mortality. Pediatrics.

[2] Edmond KM, Kirkwood BR, Amenga-Etego S, Owusu-Agyei S, Hurt LS (2007). Effect of early infant feeding practices on infection-specific neonatal mortality: an investigation of the causal links with observational data from rural Ghana. Am J Clin Nutr.

[3] Mullany LC, Katz J, Li YM, Khatry SK, LeClerq SC, Darmstadt GL, Tielsch JM (2008). Breast-feeding patterns, time to initiation, and mortality risk among newborns in southern Nepal. J Nutr.

[4] Bhutta ZA, Das JK, Rizvi A, Gaffey MF, Walker N, Horton S, Webb P, Lartey A, Black RE, Group TLNIR (2013). Evidence-based interventions for improvement of maternal and child nutrition: what can be done and at what cost?. Lancet.

[5] Black RE, Victora CG, Walker SP, Bhutta ZA, Christian P, De Onis M, Ezzati M, Grantham-McGregor S, Katz J, Martorell R (2013). Maternal and child undernutrition and overweight in low-income and middle-income countries. Lancet.

[6] Arifeen S, Black RE, Antelman G, Baqui A, Caulfield L, Becker S (2001). Exclusive breastfeeding reduces acute respiratory infection and diarrhea deaths among infants in Dhaka slums. Pediatrics.

[7] Bachrach VRG, Schwarz E, Bachrach LR (2003). Breastfeeding and the risk of hospitalization for respiratory disease in infancy: a meta-analysis. Arch Pediatr Adolesc Med.

[8] Jones G, Steketee RW, Black RE, Bhutta ZA, Morris SS, Group BCSS (2003). How many child deaths can we prevent this year?. Lancet.

[9] Iliff PJ, Piwoz EG, Tavengwa NV, Zunguza CD, Marinda ET, Nathoo KJ, Moulton LH, Ward BJ, Humphrey JH, Group ZS (2005). Early exclusive breastfeeding reduces the risk of postnatal HIV-1 transmission and increases HIV-free survival. Aids.

[10] Lewycka S, Mwansambo C, Kazembe P, Phiri T, Mganga A, Rosato M, Chapota H, Vergnano S, Newell M-L, Osrin D (2010). A cluster randomised controlled trial of the community effectiveness of two interventions in rural Malawi to improve health care and to reduce maternal, newborn and infant mortality. Trials.

[11] Kramer M, Kakuma R (2002). Optimal duration of exclusive breastfeeding (Review). Cochrane Database Syst Rev.

[12] Macro O (2005). Tanzania Demographic and Health Survey 2004–2005.

[13] Macro I (2011). Tanzania Demographic and Health Survey 2010.

[14] Adhikari M, Khanal V, Karkee R, Gavidia T (2014). Factors associated with early initiation of breastfeeding among Nepalese mothers: further analysis of Nepal Demographic and Health Survey, 2011. Int. Breastfeed. J..

[15] Cai X, Wardlaw T, Brown DW (2012). Global trends in exclusive breastfeeding. Int. Breastfeed. J..

[16] Measure D. DHS overview. ICF International. 2011. http://www.measuredhs.com/What-We-Do/Survey-Types/DHS. Accessed 15 Jan 2014.

[17] Shirima R, Greiner T, Kylberg E, Gebre-Medhin M (2001). Exclusive breast-feeding is rarely practised in rural and urban Morogoro, Tanzania. Public Health Nutr.

[18] Engebretsen IMS, Wamani H, Karamagi C, Semiyaga N, Tumwine J, Tylleskär T (2007). Low adherence to exclusive breastfeeding in Eastern Uganda: a community-based cross-sectional study comparing dietary recall since birth with 24-hour recall. BMC Pediatr.

[19] Agampodi SB, Agampodi TC, De Silva A (2009). Exclusive breastfeeding in Sri Lanka: problems of interpretation of reported rates. Int. Breastfeed. J..

[20] Mgongo M, Mosha MV, Uriyo JG, Msuya SE, Stray-Pedersen B (2013). Prevalence and predictors of exclusive breastfeeding among women in Kilimanjaro region, Northern Tanzania: a population based cross-sectional study. Int. Breastfeed. J..

[21] Mgongo M, Hashim TH, Uriyo JG, Damian DJ, Stray-Pedersen B, Msuya SE (2014). Determinants of exclusive breastfeeding in Kilimanjaro region. Tanzania Sci.

[22] Maonga AR, Mahande MJ, Damian DJ, Msuya SE (2016). Factors Affecting Exclusive Breastfeeding among Women in Muheza District Tanga Northeastern Tanzania: A Mixed Method Community Based Study. Matern Child Health J.

[23] Karkee R, Lee AH, Khanal V, Binns CW (2014). Infant feeding information, attitudes and practices: a longitudinal survey in central Nepal. Int. Breastfeed. J..

[24] Katanga J, Mgongo M, Hashim T, Stray-Pedersen B, Msuya SE (2015). Screening for Syphilis, HIV, and hemoglobin during pregnancy in Moshi municipality, Tanzania: How is the health system performing. Science.

[25] Machines IB (2013). IBM SPSS Statistics for Windows, Version 22.

[26] Mekuria G, Edris M (2015). Exclusive breastfeeding and associated factors among mothers in Debre Markos, Northwest Ethiopia: a cross-sectional study. Int. Breastfeed. J..

[27] Kearns A, Hurst T, Caglia J, Langer A, Kearns A, Hurst T, Caglia J, Langer A (2014). Focused Antenatal Care in Tanzania: Delivering Individualised, Targeted, High-quality Care.

[28] Kamudoni P, Maleta K, Shi Z, De Paoli M, Holmboe‐Ottesen G (2010). Breastfeeding perceptions in communities in Mangochi district in Malawi. Acta Paediatr.

[29] Nkala TE, Msuya SE (2011). Prevalence and predictors of exclusive breastfeeding among women in Kigoma region, Western Tanzania: a community based cross-sectional study. Int. Breastfeed. J..

[30] Asfaw MM, Argaw MD, Kefene ZK (2015). Factors associated with exclusive breastfeeding practices in Debre Berhan District, Central Ethiopia: a cross sectional community based study. Int. Breastfeed. J..

